# Effects of Saccharomyces cerevisiae fermentation product (SCFP) and phytogenic feed additive as alternatives to antibiotic growth promoters on pathogen mitigation, immunomodulation and production performance in commercial broiler chickens

**DOI:** 10.1016/j.psj.2025.105743

**Published:** 2025-08-27

**Authors:** Mahamudul Hasan Khan, Stephen Soren, Ruma Jas, Samiran Mondal, Joydeep Mukherjee, Manik Chandra Pakhira, Aditya Paul, Indranil Samanta, Anjan Mondal, Victor Nsereko, Guru Prasad Mandal

**Affiliations:** a Department of Animal Nutrition, India; b Department of Veterinary Parasitology, India; c Department of Veterinary Pathology, India; d Department of Veterinary Physiology, India; e Department of Avian Science, India; f Department of Veterinary Microbiology, West Bengal University of Animal and Fishery Sciences, Kolkata, India; g USAID-TRANSFORM, Cargill Inc., Bengaluru, Karnataka, India; h USAIDTRANSFORM, Cargill Inc., Minneapolis, MN 55440-9300, USA

**Keywords:** Broiler chickens, Commercial farm, PFA, Postbiotic, SCFP

## Abstract

The study was conducted to assess the effects of a postbiotic, *Saccharomyces cerevisiae* fermentation product (SCFP) and a commercial phytogenic feed additive (PFA) as alternative to antibiotic growth promoter(AGP) on growth performance, gut microbiota, intestinal morphology, and immune response of commercial broiler chickens at a Food and Agriculture Organization (FAO) classified sector 3 commercial poultry farm. One-day-old mixed sex Vencobb 430 Y chicks (n=4800) were randomly divided into four dietary treatment groups for 35 days experimentation encompassingT1 (positive control, PC) basal diet supplemented with bacitracin methylene disalicylate as an AGP at 500 g/MT; T2 (negative control, NC) basal diet without a growth promoter; T3 basal diet supplemented with commercial PFA at 500 g/MT; T4 basal diet supplemented with SCFP at 1.25 kg/MT.At day 35, ADG and FCR were significantly improved in PFA and SCFP groups compared to the NC group. The levels of blood glucose, total protein, albumin, triglyceride, cholesterol, and uric acid in serum showed no significant variation across different dietary treatments. From day 21 of the study, log cfu count and DNA concentration of pathogens (Enterohaemorrhagic *Escherichia coli, Salmonella*) decreased significantly in the AGP, PFA, and SCFP groups compared to the NC group. Whereas the log cfu count and average DNA concentration of *Lactobacillus* was significantly increased in PFA and SCFP groups, but not in AGP group. Increased *Lactobacillus* concentration in PFA and SCFP groups downregulated the virulence gene expression of pathogens (Enterohaemorrhagic *E. coli* and *Salmonella*). The duodenal and jejunal villi height was significantly higher in PFA and SCFP groups, although ratio of villi height to crypt depth in the duodenum, jejunum and ileum did not show significant differences among the groups. On day 28, antibody titers against the IBDV and NDV were significantly higher in SCFP groups than other groups. The present study did not identify any modulation of the cell mediated immune response in the chickens. In conclusion, addition of SCFP at 1.25 kg/MT or PFA at 500 g/MT in diet could produce better nutrient utilization ability, reduction of pathogens and their virulence gene expression, increased *Lactobacillus* count, but incorporation of SCFP increased antibody titre against two fatal viral infections such as NDV and IBDV which was not detected with PFA.

## Introduction

Maintenance of poultry gut health is a prerequisite for optimum production. Gut health is predominantly influenced by the microbial community residing in the digestive tract since the microbiome facilitates digestion and nutrient absorption, suppress infections, and train the poultry immune system for maturity(Borda-Molina et al., 2018). Several stressors present in the broiler production cycle during the post-hatching phase can alter the microbial homeostasis and increase pathogen colonization which not only compromises production but also generates the end products as a source of zoonotic and food-borne pathogens for the consumers (Attia et al., 2025; Salahi et al., 2025).

The sector 3 poultry farms are semi-commercial in nature with low to medium level biosecurity (FAO, 2010). In low-and-middle income countries like India contract broiler farms exists with low biosecurity ‘open’ sheds which are concrete/bamboo made, asbestos roofed and prone to pests like rats (Hennessey et al., 2025). Hence biosecurity management is a challenge for sector 3 poultry farms in India. Consequently, antibiotics are still considered as a risk mitigation strategy in such farms to compensate for the low biosecurity practices due to easy availability of the antibiotics, lack of stringent regulations or monitoring of the regulations (Hennessey et al., 2025).

Continuous use of antibiotics in sub-therapeutic doses may select for resistant bacterial strains in the birds which may be disseminated into the consumers creating therapeutic difficulties (Gupta et al., 2021). Hence an urgent need to develop alternative approaches for poultry farmers is necessitated to mitigate the risk of antibiotic resistant bacteria dissemination through poultry end products.

Postbiotics such as yeast fermentation products, specifically the *Saccharomyces cerevisiae* fermentation products (SCFP), appear to impact enteric pathogens across multiple livestock species. *Saccharomyces cerevisiae* fermentation products can modulate the immune system and reduce the susceptibility of birds to infections(Lensing et al., 2012). Studies with addition of SCFP in dietary rations indicate decreased *Salmonella enterica* loads and fecal shedding, decreased in *Salmonella* colonization of the lymph nodes, and reduced expression of virulence in *Salmonella* in poultry when compared to non- supplemented diets (Feye et al., 2016). Broiler chickens in a commercial farm supplemented with SCFP (0.62 kg/MT) showed reductions in both *Salmonella* virulence as expressed by *hilA* % over controls, and improved susceptibility of *Salmonella* colonies to selected common antibiotics (Potter, 2019).

Phytogenic feed additives (PFA), known as phytogenics, phytobiotics or botanicals are complex mixtures of volatile molecules (essential oils, oleoresins, and flavonoids) and their active principles, produced by the secondary metabolism of aromatic and medicinal plants. The PFA are well-recognized for antimicrobial activities, dietary immune stimulation through modulation of cytokine expression (Hassan and Awad, 2017), and overall growth of the birds with or without affecting FCR (Khattak et al., 2014). Earlier study with different essential oils like cinnamon bark oil (CNO), clove bud oil (CLO) and ajwain seed oil (AJO) revealed antibacterial property of CNO against *Escherichia coli* and was found to be more effective than an antibiotic growth promoter due to the combined beneficial effects on immune response, gut health, antioxidant status and blood cholesterol in broiler chickens (Chowdhury et al., 2018a, 2018b). Addition of PFA can increase gut beneficial bacterial growth such as *Lactobacillus* or butyrate producing *Clostridium* although the experimental results are variable ranged from neutral/no effect (Cross et al., 2007) to beneficial (Perricone et al., 2020), depending on the composition and dosage/inclusion level of PFA. Moreover, in addition to the reduction of pathogen load, direct inclusion of essential oils in diet were also found to down regulate the virulence gene expression of food-borne pathogens such as *E. coli* and *Staphylococcus* (Erfan and Marouf, 2019).

It is pertinent to evaluate the potentiality of postbiotic or PFA for modulation of gut microbiome and immune system with holistic bird growth specially in a low biosecurity farm instead of the experimental shed, which itself acts as a natural pathogen challenge for the birds. Therefore, the study was undertaken in a sector 3 commercial poultry farm to evaluate the effects of postbiotic SCFP and a commercial PFA as alternatives to an antibiotic growth promoter on the growth performance, gut health and immune responses of commercial broiler chickens.

## Materials and methods

### Study location

The study was conducted by West Bengal University of Animal and Fishery Sciences, Kolkata, West Bengal, India. The animal trial was conducted in a sector 3 commercial broiler farm (MCO Hatchery Pvt. ltd., Salkia, Howrah, West Bengal, India) with low to medium biosecurity in West Bengal, India.

### Experimental design and management of chickens

Four thousand eight hundred (n=4800) one-day-old mixed sex Vencobb 430 Y broilers were randomly divided into four dietary treatment groups. Each group consisted of four replicates, with each replicate containing 300 broiler chickens. The dietary groups were as follows- T1 (positive control, PC): Maize-soybean based diet supplemented with bacitracin methylene disalicylate (BMD, containing 100 g BMD/kg product, Zoetis India Ltd., Mumbai, India) at 500 g/MT (AGP), T2 (negative control, NC): Maize-soybean based diet without a growth promoter. T3: T2 supplemented with PFA (®PhytoGrow, a proprietary product containing cinnamon oil, turmeric oleoresin and organic acids) (The Himalaya Drug Company, Bangaluru, India) at manufacturer’s recommended dosage of 500 g/MT, T4: T2 supplemented with SCFP (Diamond™ V Original XPC™) at manufacturer’s recommended dosage of 1.25 kg/MT. The SCFP is a proprietary *Saccharomyces cerevisiae* fermentation product composed of numerous beneficial metabolites (protein, peptides, antioxidants, organics acids, nucleotides, vitamins, and minerals), beta-glucans, and mannan oligosaccharides. The experimental duration was 35 days, during which the chickens had unrestricted access to feed and water. The housing temperature, lighting, and flock density were maintained according to industry standards consistent with the commercial poultry industry. The basal diet composition and nutrition level followed the standard industry guideline (Venkys, 2017) (Table 1). The experimental diets were prepared at a feed mill on a commercial broiler farm. The additives (BMD, PFA and SCFP) were weighed and then mixed with 1 kg of the respective diets in a small capacity mechanical blender. These mixtures were added to the remainder of the diet in a mixer and thoroughly mixed to ensure proper blending of the ingredients. Care was taken to prevent cross contamination of BMD with other treatment diets during feed preparation in the feed mill. Composite feed samples from each experimental diet were collected and stored at -20°C until they were submitted for proximate analysis. Chickens were vaccinated for Newcastle Disease virus (NDV) on days 5 and 21, and for infectious bursal disease virus (IBDV) on day 12.

**Table 1:** Ingredient and nutrient composition of basal diets.

SL. No.	Ingredients ( %)	Starter (1-14 d)	Grower (15-28 d)	Finisher (29-35 d)
1	Maize	60.710	64.603	67.227
2	Soyabean meal DOC hypro	31.655	27.480	23.865
3	Maize gluten meal, 60 %	2.000	2.000	2.00
4	Soybean oil	1.139	2.100	3.286
5	Dicalcium phosphate	2.297	1.554	1.423
6	Limestone powder	0.780	0.954	0.946
7	Salt	0.286	0.279	0.281
8	DL-methionine	0.267	0.245	0.211
9	L-lysine HCL	0.310	0.236	0.213
10	L-threonine	0.085	0.069	0.069
11	Toxin binder^1^	0.100	0.050	0.050
12	Sodium bi-carbonate	0.100	0.200	0.200
13	Choline chloride, 60 %	0.100	0.075	0.075
14	Trace mineral premix^2^	0.070	0.050	0.050
15	Vitamin premix^3^	0.035	0.030	0.030
16	Coccidiostate^4^	0.050	0.050	0.050
17	Antioxidant^5^	0.015	0.015	0.015
18	Phytase^6^	0.000	0.010	0.010
**Nutrient composition**				
1	Metabolizable energy (kcal/kg)	2950.00	3100.00	3200.00
2	Crude protein ( %)	22.50	20.83	19.20
3	Ether extract ( %)	4.12	5.15	6.37
4	Crude fiber ( %)	2.74	2.66	2.57
5	Calcium ( %)	0.94	0.91	0.87
6	Available phosphorus ( %)	0.45	0.42	0.39
7	Digestible lysine ( %)	1.25	1.09	0.98
8	Digestible methionine ( %)	0.57	0.53	0.48
9	Digestible methionine + cysteine ( %)	0.94	0.88	0.80
10	Digestible threonine ( %)	0.77	0.70	0.65

^1^Mycosorb A+, Alltech Biotechnology Pvt. Ltd. Bangalore, India.

^2^Organomin Forte (contain zinc(as proteinate) min.60g/kg, iron(as proteinate) min. 30g/kg, copper(as proteinate)min.10g/kg, selenium(as proteinate) min.0.60g/kg, manganese(as proteinate)min.60.0g/kg, chromium(as proteinate)min.1.0g/kg, iodine(as potassium iodide)min.4.0g/kg), Zeus Biotech Pvt. Ltd., Mysore, India).

^3^Rovimix (each kg containsvitaminA, 48MIU; vitamin D_3_, 12MIU; vitamin E,100g; menadione, 8g; vitamin B_1,_12g;vitamin B_2_, 24g;vitamin B_6,_20g;vitamin B_12,_0.10g;biotin 0.40g, pantothenic acid, 60g; folic acid, 4g;niacin 160g; DSM Nutritional Products India Pvt. Ltd. Mahabubnagar, Telangana, India).

^4^Sacox (salinomycinsodium, 12 %), Huvepharma SEA(Pune) Pvt. Ltd., Pune, India.

^5^Endox, Kemin Industries, Inc., USA.

^6^quantamblue, AB Vista, Pune, India.

### Growth performance

Chicks were weighed replicate wise on day 1, 14, 28 and 35. The average daily gain (ADG) was calculated for each replicate. Feed intake was measured for each replicate, and the average daily feed intake (ADFI) was calculated by dividing the total feed intake per day in each replicate by the total number of chickens in the replicate. The feed conversion ratio (FCR), calculated as the gram feed intake per gram of body weight gain, was based on cumulative feed intake and weight gain in each replicate. Mortality of the chickens in each replicate was monitored regularly to calculate the mortality rate, if any, and post-mortem examinations were conducted to determine the cause of death. At the end of the trial, the mortality percentage for each replicate was calculated and used to adjust the BW, ADFI and FCR calculations.

### Sample collection

On day 4, 21, 28 and 35, eight chickens from each replicate were randomly selected for blood collection. Blood samples were collected without anticoagulant, and serum was harvested and stored at -20°C until analysis. Cloacal swabs (n=8/replicate) were randomly collected on days 7, 21,28 and 35. The swabs were placed in wide mouth flasks with cooling gel and transported to the laboratory and were processed on the same day for bacteriological study.

### Serum biochemical, hormone analyses and immune status

Concentrations of serum metabolites (glucose, total protein, albumin, uric acid, triglycerides, cholesterol) were measured on day 35 using commercial kits (Greiner Diagnostic GmbH, Germany). Concentrations of corticosterone were determined by a commercial ELISA kit (DRG Diagnostics, Germany). Antibody titers against NDV and IBDV on day 4, 21, 28 and 35 were measured using ELISA kits (IDEXX Laboratories, Inc, USA). Cell-mediated immunity was assessed by *in vitro* lymphocyte proliferation test on day 35. The proliferative response of lymphocytes was estimated using the colorimetric MTT (tetrazolium) assay (Mosmann, 1983). Phagocytic activity of neutrophils was determined using the quantitative Nitroblue Tetrazolium (NBT) assay following the standard method (Abuharfeil et al., 1999).

### Bacterial count

The population of pathogenic /zoonotic *E. coli* (Enterohaemorrhagic *E. coli,*)*, Salmonella, Clostridium, Campylobacter, Lactobacillus* and *Bifidobacterium,* in the cloacal swabs on days 7, 21, 28 and 35 was determined according to standard procedures. One gram of the caecal content was serially diluted 10-fold with sterile phosphate buffered saline (PBS), 10 μL was placed on sorbitol-MacConkey agar (for EHEC, HiMedia, India), Xylose Lysine Deoxycholate agar (for *Salmonella*, HiMedia, India), Clostridial agar (for *Clostridium*), *Lactobacillus* agar and *Bifidobacterium* agar (with supplement, HiMedia, India). The Clostridial agar plates were incubated in an anaerobic jar with gas pack (HiMedia, India). The plates were then incubated at 37°C for 24 or 48 hours, and characteristic colonies for each bacterial population were enumerated using a colony counter and the numbers were expressed as Log10 colony-forming units (CFUs) per gram of sample.

### Morphology of small intestine

On day 35, eight chickens (four males and four female) per replication were randomly selected and euthanised. Sections measuring 2-3 cm of the duodenum, jejunum (between the entry of the bile duct and Mackel’s diverticulum) and ileum were extracted, rinsed with PBS and preserved in 10 % formalin. Tissue sections were processed using standard histopathological procedures (Kumar et al., 2017) and measurements of villi height and crypt depth were conducted using an ocular microscope. The height (µm) from the tip of the villus to the villus–crypt junction was considered as villus height (VH) and the depth of the invagination between two villi was considered as crypt depth (CD). Three sections with 10 observations each were analysed for every sample and the mean values (µm) were used to derive a single observation.

### Antibiogram, PCR based detection of antibiotic resistance genes (ESBL and *bac*) and nucleotide sequencing

The antibiogram of EHEC isolates was performed using the disc diffusion method with commonly used antibiotics in poultry following the CLSI guidelines (CLSI, 2017). PCR was conducted to detect major extended spectrum beta-lactamase (ESBL) genes (*bla_CTX-M-Type_, bla_TEM-Type_, bla_SHV-Type_*) and bacitracin resistance gene (*bac*) according to standard protocols (Weill et al., 2004; Adimpong et al., 2012). Nucleotide sequencing of selected PCR products targeting ESBLgenes was carried out by a commercial enterprise (NalgenBio, India) to determine the ESBL gene subtypes.

### Quantification of bacterial DNA

DNA was extracted from selected *E. coli, Salmonella, Bifidobacterium* and *Lactobacillus* strains isolated from positive control (T1) and treatment groups (T2, T3, T4) using a commercial DNA extraction kit (DNeasy Power Lyzer Microbial Kit, Qiagen). Quantification of extracted bacterial DNA was conducted in Nanodrop Biospectrophotometer (Eppendorf, Germany).

### Real-time PCR based detection of virulence gene expression down regulation

After the extraction of bacterial RNA using commercial kit (PureZol, BioRad, USA) from selected pathogenic/zoonotic *E. coli* (EHEC) and *Salmonella* strains isolated from the treatment groups of the chickens were assessed for the downregulation of selected virulence gene (*invA* for *Salmonella* and *eaeA* for EHEC) expression in comparison to the isolates of the positive control group (T1).

The concentration of isolated RNA samples was determined using Nanodrop Biospectrometer (Eppendorf, Germany). The quality of the RNA samples was analysed by running RNA samples in denaturing agarose gel electrophoresis. The first strand cDNA was synthesized from the isolated total RNA. Reverse transcription was carried out in 20 µl reaction mixtures. Constant amount (1 µg) of RNA was reverse transcribed using 4 µl of iScript Reverse Transcriptase cDNA synthesis kit (BioRad, USA) as per the manufacturer’s instruction. The cDNA thus synthesized was stored at -20 °C for RT-qPCR for determining the expression of virulence genes of EHEC (*eaeA*) and *Salmonella* (*invA*). The *gapA* and GAPDH genes were selected as housekeeping genes of *E. coli* and *Salmonella*, respectively. Validation of cDNA and end point PCR conditions were optimized to amplify the housekeeping as well as virulence genes of *E. coli* and *Salmonella* in gradient thermal cycler (BioRad, USA) using the primers described earlier (Jandu et al., 2009; Kendall et al., 2012; Kumar and Mondal, 2015). The annealing temperature was standardized using cDNA by performing gradient PCR.

Real-time PCR was performed (CFX touch, BioRad, USA) operational with BioRad CFX manager using qPCR kit (BioRad, USA). The qPCR master mix (SSO Fast Eva Green ®, BioRad, USA) was strictly protected from exposure to light during the entire mixing process. The relative expression of *eaeA* gene of *E. coli* and *invA* gene of *Salmonella* isolated from the experimental chickens was studied by performing RT-qPCR and semi quantitative gene expression analysis. The results were normalized to housekeeping gene of both bacteria species relative to the positive control group of chickens (T1). RT-qPCR data were obtained as average CT value and the fold change expression of *eaeA* gene of *E. coli*, and *invA* gene of *Salmonella* in terms of 2^^-∆∆ct^value were calculated as per the standard method (Pfaffl, 2001).

### Statistical analysis

Individual replicate was used as the experimental unit for body weight gain, feed intake and FCR, while individual bird was treated as experimental unit for other parameters. A Shapiro-Wilk’s test (Shapiro and Wilk, 1965) and a visual inspection of the histograms showed that data were approximately normally distributed. Data were analysed by one-way analysis of variance (ANOVA)using SPSS software (SPSS, 2011). To compare the effect of diets, GLM procedure was performed. If differences (P<0.07) were noted, Duncan's Multiple Range Test (DMRT)was used to compare the means. P-values <0.05 were considered statistically significant and P-values <0.07 were considered trend.

## Results

### Growth performance

Growth performance of broiler chickens is presented in Table 2. The ADG of the chickens significantly increased in the T1, T3 and T4 groups compared to the T2 group from1 to 14 (P=0.008) and 1-35 days (P= 0.012) of age. The T1, T3 and T4 groups also had significantly higher final body weights compared to the T2 group (P=0.014). However, there were no significant (P<0.05) differences in ADG during the 15-28 days and 29-35 days periods. There were no significant differences in ADFI among the treatment groups (p>0.05). FCR of the chickens improved significantly in the T1, T3 and T4 groups compared to the T2 group from 1-14 days of age (P= 0.008). During, 15-28 days of age, compared to T2 group, FCR was tended to improve in the T3 and T4 groups (P=0.061). However, there were no significant differences in the FCR among the treatment groups during the finisher phase (29-35 days). Throughout the entire period (1-35 days), FCR was significantly improved (P= 0.011) in the T3 and T4 groups compared to the T2 group, although no significant differences were seen among the T1, T2 and T3 groups. Additionally, there were no significant differences in bird mortality ( %) among the dietary treatment groups (P>0.05).

**Table 2:** Effect of phytogenic feed additive and postbiotic (Saccharomyces cerevisiae fermentation products) on final body weight (BW), average daily gain (ADG), average daily feed intake (ADFI) and feed conversion ratio (FCR) and mortality of broiler chickens.

Attribute^1^	Treatment				SEM	P-Value
	T1	T2	T3	T4		
ADG (g/d)						
1-14 d	27.97^a^	26.20^b^	27.88^a^	27.80^a^	0.24	0.008
15-28 d	63.76	61.33	63.16	64.45	0.52	0.172
29-35 d	61.68	62.80	63.60	65.16	1.04	0.726
1-35 d	49.03^a^	47.57^b^	49.14^a^	49.93^a^	0.29	0.012
Final BW (g)	1756.25^a^	1705.87^b^	1759.80^a^	1787.85^a^	10.05	0.014
ADFI (g/d)						
1-14 d	30.67	31.80	31.17	30.51	0.27	0.329
15-28 d	89.73	89.54	87.06	89.58	0.59	0.333
29-35 d	115.84	108.25	109.42	106.69	1.74	0.274
1-35 d	71.33	70.19	69.18	69.38	0.48	0.405
FCR (g intake/g gain)						
1-14 d	1.10^b^	1.22^a^	1.12^b^	1.10^b^	0.02	0.008
15-28 d	1.41^ab^	1.46^a^	1.38^b^	1.39^b^	0.02	0.061
29-35 d	1.88	1.73	1.73	1.64	0.04	0.152
1-35 d	1.46^ab^	1.48^a^	1.41^b^	1.39^b^	0.01	0.011
Mortality ( %)	3.83	4.34	3.83	4.25	0.16	0.591

^abc^means bearing different superscripts in the same row differ significantly (p ≤ 0.05).

^+^T1: Maize-soybean meal-based diet supplemented with BMD at 500 g/MT feed; T2: Maize soyabean meal-based diet without growth promoters; T3: T2 diet supplemented with phytogenic feed additive at 500 g/MT feed; T4: T2 diet supplemented with *Saccharomyces cerevisiae* fermentation products (SCFP) at 1.25 kg/MT feed; SEM: Total standard error of means.

^1^Means are based on 4 replicates per treatment (n=4).

### Blood biochemical profile

As shown in Table 3, no significant variation (p > 0.05) was observed in the levels of glucose, total protein, albumin, triglyceride, cholesterol, and uric acid in serum across the various dietary treatments. Corticosterone concentration was significantly lower (P= 0.008) in the T3 and T4 groups compared to the T1 and T2 groups.

**Table 3:** Effect of phytogenic feed additive and postbiotic (Saccharomyces cerevisiae fermentation products) on blood biochemical profile serum cortisol concentration in broiler chickens at day 35.

Attribute^1^	Treatment				SEM	P-Value
	T1	T2	T3	T4		
Glucose (mg/dl)	165.38	160.63	171.00	166.00	4.65	0.901
Total Protein(mg/dl)	2.59	2.73	2.88	2.85	0.09	0.642
Albumin(mg/dl)	1.83	1.79	2.16	1.93	0.06	0.109
Triglyceride (mg/dl	50.75	63.88	46.75	55.75	3.20	0.269
Cholesterol(mg/dl))	78.39	92.37	99.13	84.63	3.35	0.138
Uric Acid(mg/dl)	3.99	3.94	3.50	3.24	0.14	0.166
Corticosterone(nmol/L)	2.05^a^	2.03^a^	1.82^b^	1.88^b^	0.03	0.008

^ab^means bearing different superscripts in the same row differ significantly (p ≤ 0.05).

^+^T1: Maize-soybean meal-based diet supplemented with BMD at 500 g/MT feed; T2: Maize soyabean meal-based diet without growth promoters; T3: T2 diet supplemented with phytogenic feed additive at 500 g/MT feed; T4: T2 diet supplemented with *Saccharomyces cerevisiae* fermentation products (SCFP) at 1.25 kg/MT feed; SEM: Total standard error of means.

^1^Means are based on 8 replicates per treatment (n=8).

### Gut bacteria

The viable bacteria numbers (log_10_ CFU/g) in cloacal swab in broiler chickens are presented in Table 4. On day 7, the counts of Enterohaemorrhagic *E. coli* (EHEC) (P= 0.040) and *Campylobacter* (P= 0.007) were significantly lower in T1, T3 and T4 groups compared to the T2 group. The count of *Salmonella* was significantly lower in T4 group compared to the T1, T2 and T3 groups (P=0.001). Additionally, the count of *Lactobacillus* was significantly higher in T4 group compared to the T1, T2 and T3 groups (P=0.005). However, there were no significant differences among the dietary treatment groups in the counts of *Clostridium* and *Bifidobacterium* (P>0.05). On day 21, the count of EHEC was significantly lower in T3 and T4 groups compared to the T1 and T2 groups (P=0.000). The count of *Salmonella* was significantly lower in T1, T3 and T4 groups compared to the T2 group (P=0.001). Additionally, the count of *Lactobacillus* was significantly higher in T3 and T4 groups compared to the T1 and T2 groups (P=0.000). However, there were no significant differences among the dietary treatment groups in the counts of *Clostridium, Campylobacter* and *Bifidobacterium* (P>0.05). On day 28, the count of EHEC was significantly lower in T4 group compared to the T1, T2 and T3 groups (P=0.003). The *Salmonella* count was significantly lower in the T1 and T4 groups compared to the T2 group (P= 0.045), whereas the T3 group showed no significant difference from the T1, T2, and T4 groups. Additionally, the count of *Lactobacillus* was significantly higher in T4 group compared to the T1, T2 and T3 groups (P=0.000). However, there were no significant differences in the counts of *Clostridium, Campylobacter* and *Bifidobacterium* among the dietary treatment groups (P>0.05). On day 35, the count of EHEC was significantly lower in T4 group compared to the T1, T2 and T3 groups (P=0.002). However, there were no significant differences among the dietary treatment groups in the counts of *Salmonella, Clostridium, Campylobacter, Lactobacillus* and *Bifidobacterium* (P>0.05).

**Table 4:** Effect of phytogenic feed additive and postbiotic (Saccharomyces cerevisiae fermentation products) on viable bacteria numbers (log_10_ CFU/g) in cloacal swab in broiler chickens.

Attribute^1^	Treatment				SEM	P-Value
	T1	T2	T3	T4		
**Day 7**						
*Enterohaemorrhagic E. coli*	3.69^b^	3.93^a^	3.60^b^	3.69^b^	0.04	0.040
*Salmonella*	4.24^a^	4.18^a^	4.07^a^	3.88^b^	0.04	0.001
*Clostridium*	5.40	5.38	5.18	5.24	0.03	0.053
*Campylobacter*	3.50^b^	4.04^a^	3.65^b^	3.44^b^	0.07	0.007
*Lactobacillus*	5.52^b^	5.43^b^	5.65^b^	6.09^a^	0.07	0.005
*Bifidobacterium*	5.50	5.29	5.55	5.59	0.05	0.142
**Day 21**						
*Enterohaemorrhagic E. coli*	4.37^b^	4.55^a^	4.17^c^	4.07^c^	0.04	0.000
*Salmonella*	3.40^b^	4.48^a^	4.20^b^	4.09^b^	0.05	0.001
*Clostridium*	5.15	5.25	5.16	5.30	0.03	0.063
*Campylobacter*	4.54	4.37	4.22	4.27	0.05	0.115
*Lactobacillus*	5.14^c^	5.37^b^	5.64^a^	5.58^a^	0.05	0.000
*Bifidobacterium*	5.29	5.52	5.37	5.35	0.03	0.112
**Day 28**						
*Enterohaemorrhagic E. coli*	4.34^a^	4.18^a^	4.39^a^	3.87^b^	0.06	0.003
*Salmonella*	6.77^b^	7.26^a^	7.17^ab^	6.77^b^	0.08	0.045
*Clostridium*	5.43	5.39	5.28	5.36	0.13	0.114
*Campylobacter*	4.84	5.19	5.27	5.17	0.06	0.071
*Lactobacillus*	6.13^c^	6.33^bc^	6.41^b^	6.70^a^	0.05	0.000
*Bifidobacterium*	6.24	6.19	6.38	6.66	0.07	0.074
**Day 35**						
*Enterohaemorrhagic E. coli*	4.37^a^	4.49^a^	4.46^a^	4.22^b^	0.03	0.002
*Salmonella*	6.14	5.97	5.94	6.17	0.07	0.632
*Clostridium*	4.08	4.49	4.10	4.28	0.07	0.085
*Campylobacter*	5.91	5.64	5.74	5.88	0.06	0.233
*Lactobacillus*	5.41	5.61	5.17	5.24	0.07	0.106
*Bifidobacterium*	5.30	5.50	5.81	5.50	0.10	0.319

^abc^means bearing different superscripts in the same row differ significantly (p ≤ 0.05).

^+^T1: Maize-soybean meal-based diet supplemented with BMD at 500 g/MT feed; T2: Maize soyabean meal-based diet without growth promoters; T3: T2 diet supplemented with phytogenic feed additive at 500 g/MT feed; T4: T2 diet supplemented with *Saccharomyces cerevisiae* fermentation products (SCFP) at 1.25 kg/MT feed; SEM: Total standard error of means.

^1^Means are based on 8 replicates per treatment (n=8).

### Antibiogram, PCR based detection of antibiotic resistance genes (ESBL and *bac*) and nucleotide sequencing

Enterohaemorrhagic *E. coli* isolates showed highest phenotypical resistance against erythromycin (100 %), clindamycin (100 %), cefpodoxime (100 %), cephalexin (100 %), amoxicillin (92 %), doxycycline (92 %) and enrofloxacin (92 %). The isolates showed less resistance against gentamicin (34 %) and chloramphenicol (38 %) (Table 5).

**Table 5:** Antibiogram of Enterohaemorrhagic Escherichia coli (EHEC) isolates.

Antibiotic	Resistant EHEC isolates (percentage in parenthesis)
Erythromycin (15 mcg)	26/26 (100 %)
Ceftazidime (30 mcg)	23/26 (88.4 %)
Doxycycline (30 mcg)	24/26 (92.3 %)
Enrofloxacin (10 mcg)	24/26 (92.3 %)
Cindamycin (10 mcg)	26/26 (100 %)
Gentamicin (10 mcg)	9/26 (34.6 %)
Cefpodoxime (10 mcg)	26/26 (100 %)
Amoxicillin (10 mcg)	24/26 (92.3 %)
Chloramphenicol (25 mcg)	10/26 (38.4 %)
Cephalexin (30 mcg)	26/26 (100 %)

In PCR, majority of the EHEC isolates possessed *bla_TEM-Type_*, followed by *bla_SHV-Type_* and *bla_CTX-M-Type_*. None of the isolates possessed *bac* gene. The BLAST analysis of nucleotide sequences revealed presence of TEM-1 and CTX-M-15.

### Quantification of bacterial DNA

**Fig. 1:**
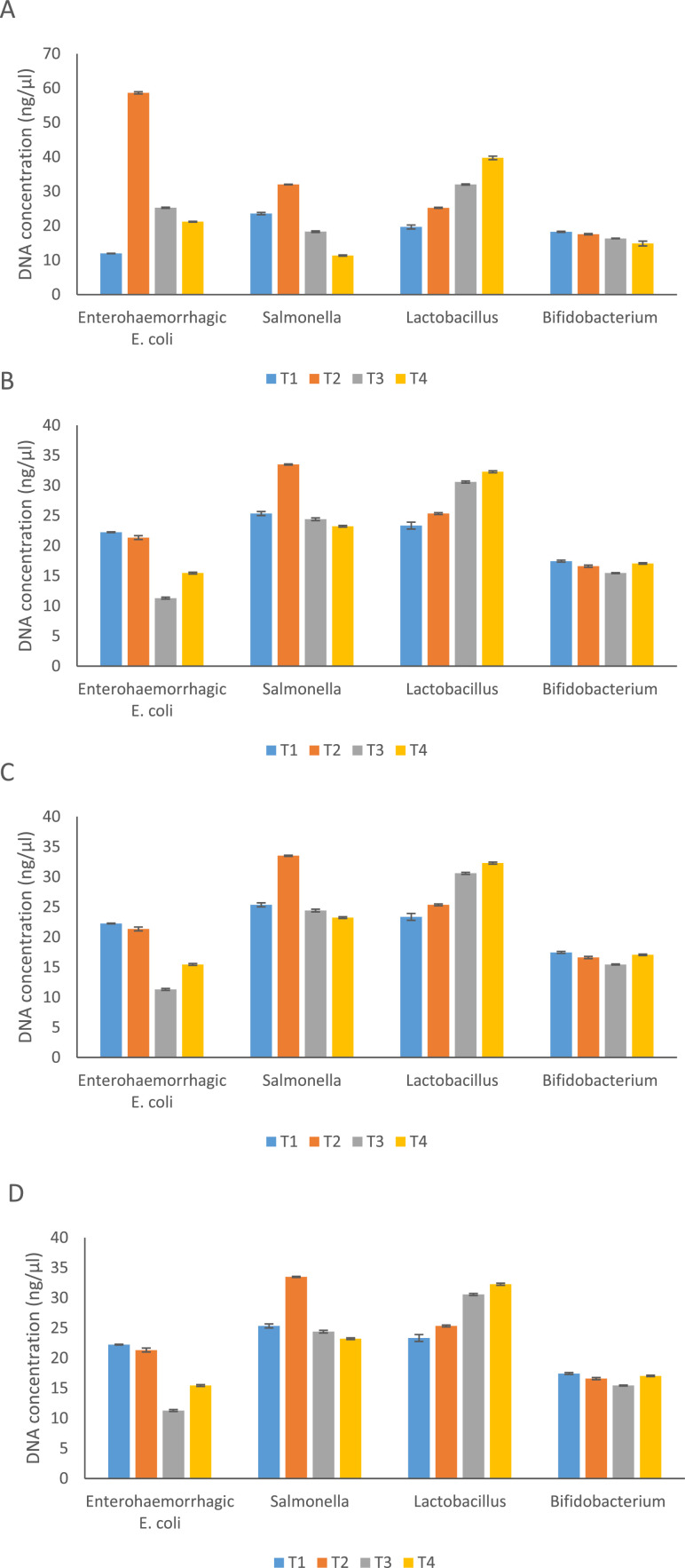
Effect of phytogenic feed additive and postbiotic (Saccharomyces cerevisiae fermentation products) on bacterial DNA concentration (ng/µl) in cloacal swab in broiler chickens on day 7 (A), 21 (B), 28 (C) and 35 (D).

On day 7, similar to bacterial count, bacterial genomic DNA concentration for EHEC were significantly lower in T1, T3 and T4 groups compared to the T2 group (P=0.000) (Fig. 1). The average bacterial DNA concentration of *Salmonella* was significantly lower in T4 group compared to the T1, T2 and T3 groups (P= 0.000). Additionally, the DNA concentration of *Lactobacillus* was significantly higher in T4 group compared to the T1, T2 and T3 groups (P=0.000)). On day 21, similar to bacterial count, DNA concentration of EHEC was significantly lower in T3 and T4 groups compared to the T1 and T2 groups (P=0.000). The DNA concentration of *Salmonella* was significantly lower in T1, T3 and T4 groups compared to the T2 group (P=0.000). Additionally, the DNA concentration of *Lactobacillus* was significantly higher in T3 and T4 groups compared to the T1 and T2 groups (P=0.000). On day 28, the DNA concentration of EHEC was significantly lower in T4 group compared to the T1, T2 and T3 groups (P=0.000). The DNA concentration of *Salmonella* was significantly lower in T1 and T4 groups compared to the T2 group (P=0.000). Additionally, the DNA concentration of *Lactobacillus* was significantly higher in T4 group compared to the T1, T2 and T3 groups (P=0.034). On day 35, the DNA concentration of EHEC and *Salmonella* was significantly lower in T4 group compared to the T1, T2 and T3 groups (P= 0.000). The DNA concentration of *Lactobacillus* was significantly T2, T3 and T4 groups compared to T1 group (P= 0.000).

### Real-time PCR based detection of virulence gene expression down regulation

**Fig. 2:**
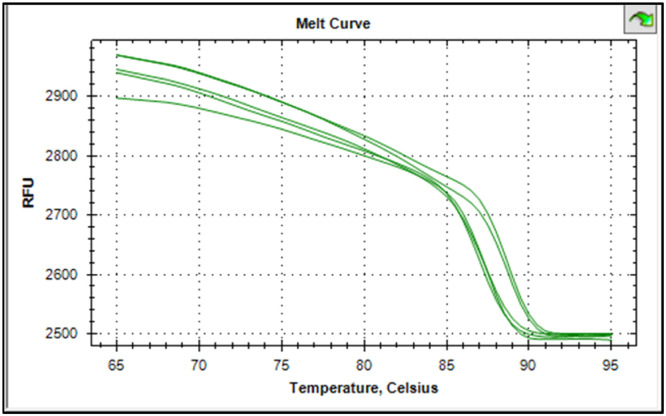
Showing melting curve of gapA gene of E. coli.

**Fig. 3:**
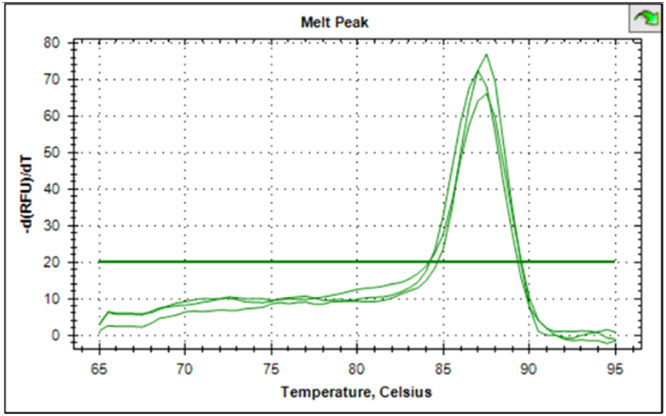
Showing the melting peak of gapA gene of E. coli.

**Fig. 4:**
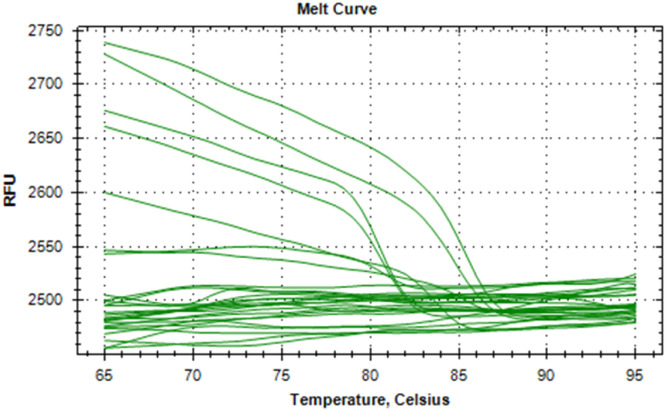
Showing melting curve of eae gene of E. coli.

**Fig. 5:**
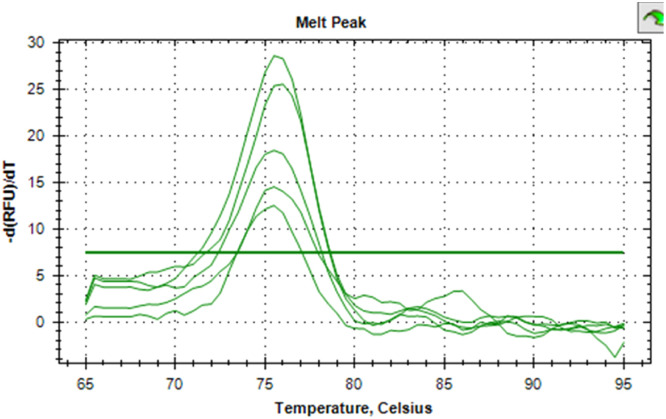
Showing the melting peak of eae gene of E. coli.

**Fig. 6:**
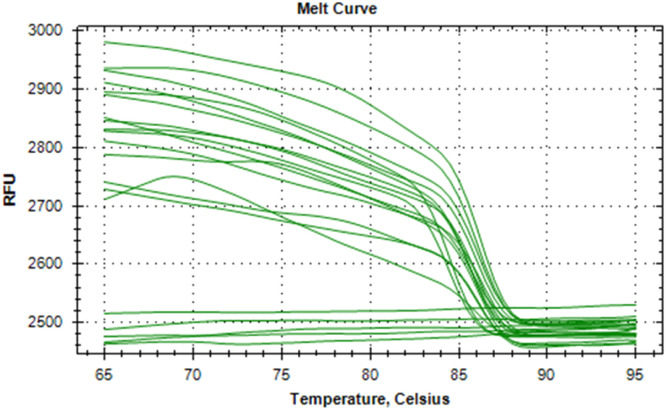
Showing melting curve of gapdh gene of Salmonella.

**Fig. 7:**
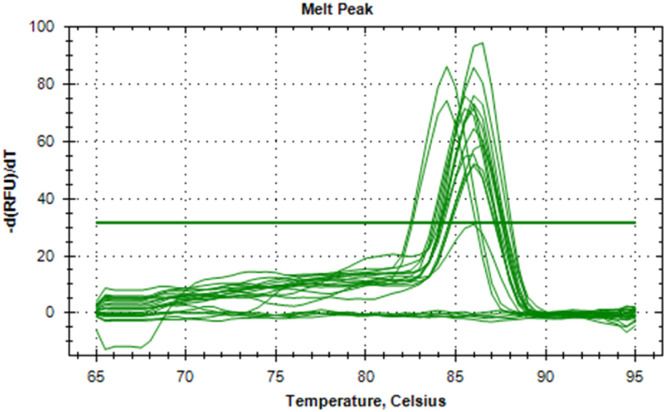
Showing the melting peak of gapdh gene of Salmonella.

**Fig. 8:**
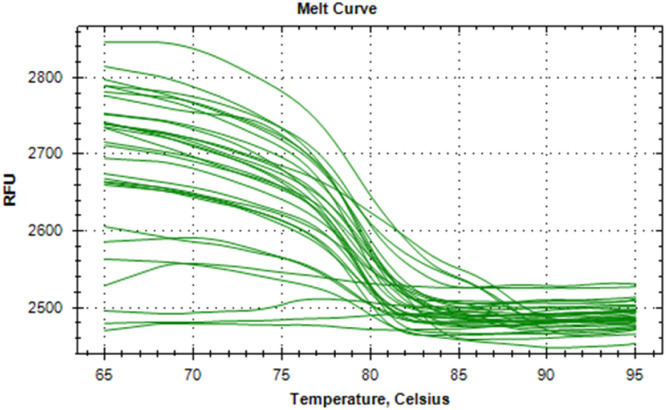
Showing melting curve of invA gene of Salmonella.

**Fig. 9:**
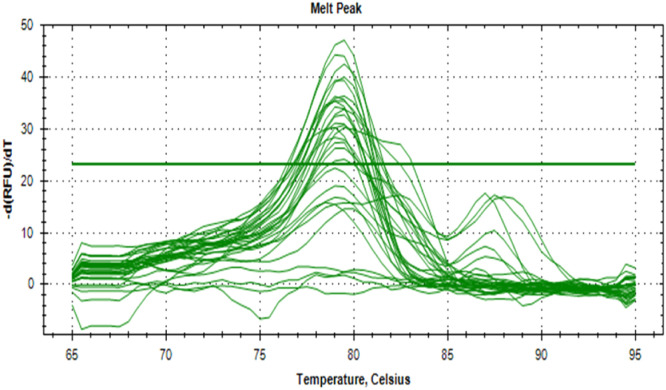
Showing the melting peak of invA gene of Salmonella.

The synthesized cDNA was validated by amplifying housekeeping genes using specific markers. For accurate gene expression studies, it is mandatory to normalize the results of RT-qPCR experiments using one housekeeping gene. In the present study, *gapA* gene of *E. coli* and *gapdh* gene of *Salmonella* were tested to normalize the data obtained for the virulence genes (*eaeA* gene of *E. coli* and *invA* gene of *Salmonella*) of bacteria. The relative semi-quantitative expression of target genes (*eaeA* gene of *E. coli* and *invA* gene of *Salmonella*) in terms of fold chain were statistically compared among the four different treatment groups of chickens (Table 6). The melting curve as well as melting peak of *gapA* and *eaeA* genes of *E. coli* and *gapdh* and *invA* genes of *Salmonella* as obtained by RT-q PCR were presented (Fig 2 to 9). The relative expression of *eaeA* gene of *E. coli* was higher in T2 group in comparison to other groups but there was no significant difference (P>0.05) among them. Significant (P= 0.045) upregulation of relative expression of *invA* gene of *Salmonella* was also observed in T2 group compared to other groups.

**Table 6:** Effect of phytogenic feed additive and postbiotic (Saccharomyces cerevisiae fermentation products) on relative mRNA expression of virulent genes of E. coli and Salmonella) in cloacal swab in broiler chickens.

Attribute^1^	Treatment				SEM	P-Value
	T1	T2	T3	T4		
eae gene of *E. coli*	1.36	4.17	2.38	1.96	0.48	0.185
*invA* gene of *Salmonella*	1.46^b^	5.01^a^	2.38^b^	2.16^b^	0.50	0.045

^abc^means bearing different superscripts in the same row differ significantly (p ≤ 0.05).

^+^T1: Maize-soybean meal-based diet supplemented with BMD at 500 g/MT feed; T2: Maize soyabean meal-based diet without growth promoters; T3: T2 diet supplemented with phytogenic feed additive at 500 g/MT feed; T4: T2 diet supplemented with *Saccharomyces cerevisiae* fermentation products (SCFP) at 1.25 kg/MT feed; SEM: Total standard error of means.

^1^Means are based on 8 replicates per treatment (n=8).

Fig. 2, Fig. 3, Fig. 4, Fig. 5, Fig. 6, Fig. 7, Fig. 8, Fig. 9

### Gut morphology

In the duodenum, villi height (VH) was significantly higher in the T3 and T4 groups compared to the T2 group (P=0.034), while the T1 group showed no difference from the T2, T3 and T4 groups (Table 7). In the jejunum, VH was significantly higher in the T1, T3 and T4 groups compared to the T2 group (P= 0.031). Crypt depth (CD) in jejunum was significantly lower in the T4 group compared to the T1 and T2 groups (P= 0.024), while the T3 group did not differ from the T1, T2 and T4 groups. However, VH in the ileum, CD in the duodenum and ileum, and VH/CD ratio in the duodenum, jejunum and ileum did not show significant differences among the dietary treatment groups (P>0.05).

**Table 7:** Effect of phytogenic feed additive and postbiotic (Saccharomyces cerevisiae fermentation products) on gut morphology in broiler chickens at day 35.

Attribute^1^	Treatment				SEM	P-Value
	T1	T2	T3	T4		
**Duodenum**						
Villi height (VH; μm)	1394.08^ab^	1251.95^b^	1453.23^a^	1455.33^a^	28.98	0.034
Crypt depth (CD; μm)	132.41	128.02	128.18	127.53	5.96	0.992
VH/CD ratio	10.88	10.67	12.66	12.18	0.65	0.666
**Jejunum**						
Villi height (VH; μm)	1194.82^a^	949.62^b^	1208.27^a^	1200.85^a^	37.90	0.031
Crypt depth (CD; μm)	94.62^a^	91.72^a^	81.43^ab^	75.00^b^	2.67	0.024
VH/CD ratio	13.04	12.97	14.86	16.43	0.64	0.627
**Ileum**						
Villi height (VH; μm)	743.12	688.75	822.08	737.85	28.38	0.435
Crypt depth (CD; μm)	75.85	73.60	82.70	77.15	3.16	0.789
VH/CD ratio	10.15	9.53	10.25	9.98	0.41	0.933

^ab^means bearing different superscripts in the same row differ significantly (p ≤ 0.05).

^+^T1: Maize-soybean meal-based diet supplemented with BMD at 500 g/MT feed; T2: Maize soyabean meal-based diet without growth promoters; T3: T2 diet supplemented with phytogenic feed additive at 500 g/MT feed; T4: T2 diet supplemented with *Saccharomyces cerevisiae* fermentation products (SCFP) at 1.25 kg/MT feed; SEM: Total standard error of means.

^1^Means are based on 8 replicates per treatment (n=8).

### Immune response

As presented in Table 8, antibody titers against the IBD vaccine were significantly higher in the T3 and T4 groups compared to the T2 and T1 groups on day 28 (P= 0.003). However, on day4, 21 and 35, there were no significant differences (p > 0.05) among the dietary treatment groups in antibody titers against this vaccine. For the ND vaccine, on day 28, antibody titers were significantly higher in the T4 group compared to the T2 and T1 groups (P = 0.036), while the T3 group did not differ from the T1, T2 and T4 groups. On day 35, antibody titers against the ND vaccine were significantly higher in the T3 and T4 groups compared to the T2 and T1 groups (P = 0.010). However, on days 4 and 21, there were no significant differences (p > 0.05) among the dietary treatment groups in antibody titers against this vaccine. No significant differences were observed among the treatment groups in the for the vitro phagocytic activity of neutrophils and lymphocytes (P>0.05).

**Table 8:** Effect of phytogenic feed additive and postbiotic (Saccharomyces cerevisiae fermentation products) on antibody titre (log_10_) against Infectious bursal disease virus (IBDV) and Newcastle disease virus (NDV), phagocytic activity of neutrophil (as expressed in optical density at 450 nm) and lymphocytes (stimulation index) in broiler chickens.

Attribute^1^	Treatment				SEM	P-Value
	T1	T2	T3	T4		
Antibody titre						
IBDV-4d	2.84	3.03	2.82	2.77	0.12	0.893
IBDV-21 d	2.36	2.22	2.47	2.20	0.09	0.677
IBDV-28 d	3.20^b^	3.17^b^	3.45^a^	3.46^a^	0.04	0.003
IBDV-35 d	3.36	3.31	3.31	3.40	0.02	0.340
NDV-4 d	2.67	2.63	2.77	2.873	0.06	0.853
NDV-21 d	2.22	1.83	1.94	1.93	0.08	0.424
NDV-28 d	2.38^b^	2.46^b^	2.49^ab^	2.66^a^	0.04	0.036
NDV-35 d	2.76^b^	2.63^b^	3.14^a^	3.07^a^	0.06	0.010
In vitro phagocytic activity						
Neutrophil	0.55	0.55	0.59	0.53	0.02	0.704
Lymphocyte	0.91	0.86	0.95	1.10	0.06	0.536

^ab^means bearing different superscripts in the same row differ significantly (p ≤ 0.05).

^+^T1: Maize-soybean meal-based diet supplemented with BMD at 500 g/MT feed; T2: Maize soyabean meal-based diet without growth promoters; T3: T2 diet supplemented with phytogenic feed additive at 500 g/MT feed; T4: T2 diet supplemented with *Saccharomyces cerevisiae* fermentation products (SCFP) at 1.25 kg/MT feed; SEM: Total standard error of means.

^1^Means are based on 8 replicates per treatment (n=8).

## Discussion

Supporting the findings of the present study previous reports have shown that SCFP (Chuang et al., 2021; Linh et al., 2021; Ismael et al., 2022;Liza et al., 2022; Soren et al., 2024), PFA particularly the EOs (Chowdhury et al., 2018a; Su et al., 2021; Zhang et al., 2023) and combination of postbiotic and EO combination (Doski and Kareem, 2024) could promote growth performance in broilers. The present study conducted in a sector 3 commercial farm, data showed that flocks fed SCFP and PFA had improved ADG and FCR, which could be due to the positive effect of SCFP (Soren et al., 2024) and EO (Mountzouris et al., 2011; Attia et al., 2019) on bird’s digestive system since they help to restore the gut microbiota balance and nutrient absorption. Nonetheless, the administration of PFA (Amad et al., 2011) and SCFP (Chuang et al., 2021; Oliveira et al., 2022) as growth promoter does not always improve production performance although enhanced FCR.

The addition of SCFP in the experimental bird’s diet did not affect the concentration of serum glucose, total protein, albumin, triglyceride, cholesterol and uric acid indicating no adverse side effects on the birds (Yalçın et al., 2013). Similarly, Liza et al. (2022) found no significant differences in serum metabolites between dietary groups when incorporating 3.5 % SCFP in broiler chickens. Other studies (Liza et al., 2022; Soren et al., 2024) also reported no significant differences in serum metabolites with SCFP supplementation. In this study, SCFP and PFA supplemented groups showed significantly lower corticosterone concentration compared to other treatment groups. Similar results were reported by earlier study (Soren et al., 2024) when incorporating SCFP at 1.25 kg/MT feed in broiler chickens.

Broiler chickens provided with PFA and SCFP supplements may have a beneficial effect on the populations of gut bacteria, which in turn may improve the gut beneficial bacterial load such as butyrate producing-*Clostridium* and *Lactobacillus*, respectively (Mirza et al., 2020; Shehata et al., 2022; Sultan et al., 2024;). In this study, data on gut microbiota aligned with Soren et al. (2024) who reported that supplementing SCFP in the diet of broiler chickens significantly lower the *E. coli,* EHEC*, ESBL producing Enterobacteriaceae* and *Salmonella* counts. Similar observations were reported by Roto et al. (2017) who found that the addition of SCFP at 1.25 g/kg resulted in a significant reduction of *Salmonella* compared to the control group. Gingerich et al. (2021) also reported that the addition of SCFP at 1.5 kg/MT to poultry feed significantly reduced *Salmonella* occurrence compared to the control group. The DNA concentration of *Lactobacillus* was significantly higher in SCFP group compared to other groups. It could be explained with the finding of significantly high *Lactobacillus* count and their average DNA concentration in PFA and SCFP groups especially during day 21 and day 28. Earlier study observed a correlation between down regulation of expression of *Salmonella* virulence genes in the chicken cecum and the presence of a high population of *Lactobacillus* (Yang et al., 2014).

Enterohaemorrhagic *E. coli* isolates showed highest phenotypical resistance against common antibiotics. Similarly earlier study reported high (>65 %) resistance to erythromycin and ampicillin and sensitivity to gentamicin of the EHEC isolates from kids, lambs and calves (Medina et al., 2011). Majority of the isolates possessed *bla_TEM-Type_* gene among ESBL and none of the isolates possessed *bac* gene. Similarly, majority of the EHEC isolates possessed *bla_TEM-Type_* gene (91 %) showing resistance to ampicillin in earlier study (Medina et al., 2011). Although the poultry birds from Asian countries mostly harbour the *bla_CTX-M-Type_* gene, including the variants *bla_CTX-M-1_, bla_CTX-M-14_*, and *bla_CTX-M-15_* (Mandujano-Hernández et al., 2024), but the trend is shifting towards possession of *bla_TEM-Type_* in the poultry isolates as detected recently in China (Rizal et al., 2024).

Small intestine is essential for digestion and absorption of nutrients. The morphology of small intestine provides an indicator for its absorptive capacity. Our results showed that PFA increased villi height in duodenum and jejunum, which is similar with earlier reports (Yarmohammadi Barbarestani et al., 2020; Sue et al., 2021). The supplementation of SCFP increased VH in small intestine, indicating improved absorption (Lin et al., 2023; Soren et al., 2024). The present results align with Lin et al. (2023), who observed a significant increase in jejunal VH with dietary supplementation of *Saccharomyces cerevisiae* hydrolysate at doses of 250 mg/kg. Similarly, Chuang et al. (2021) found that including 10 % *Saccharomyces cerevisiae* fermented wheat bran in poultry diets led to a significant increase in VH, but no differences were observed in CD and VH/CD ratio among the groups studied. Soren et al. (2024) reported increased VH in the duodenum and jejunum in the SCFP supplemented group.

Antibody titre against IBDV vaccine on day 28 was greater in PFA and SCFP groups compared to positive control group. This result was in agreement with Soren et al. (2024) who reported increased antibody titre against IBDV vaccine when broiler chickens were supplemented with SCFP at 1.25 kg/MT. Moreover, the present study showed that PFA and SCFP enhanced antibody titre against NDV vaccine on day 28, and day 28 and 35, respectively, in comparison with the positive control. These findings align with Ismael et al. (2022), who reported a significant difference in antibody titres against the NDV vaccine when 0.625 kg/t of SCFP was added to the diets of broiler chickens. Cortés-Coronado et al. (2017) also reported significant results against the NDV vaccine when SCFP was tested at different concentrations. Similarly, Soren et al. (2024) found that antibody titer against NDV vaccines were significantly higher in the SCFP supplemented group. However, the present study did not identify any modulation of the cell mediated immune response in the chickens studied. This response is typically more pronounced in challenge studies, particularly with intracellular pathogens like *Coccidia*, when fed with yeast hydrolysate. Additionally, the modulation was found to be dependent on the dosage of the yeast products (Gao et al., 2009). These findings are consistent with a previous study by Soren et al. (2024), who found that supplementing the broiler diet with SCFP did not significantly affect the in vitro phagocytic activity of neutrophils and lymphocytes.

## Conclusion

The feed conversion ratio was significantly better in the AGP, PFA and SCFP groups compared to the negative control group during the entire experimental period, although the AGP added group showed no significant difference in FCR from the PFA and SCFP groups. The log cfu count and average DNA concentration of pathogens (EHEC, *Salmonella*) decreased significantly in the groups added with both antibiotics and non-antibiotic growth promoters than negative control from day 21 of the study. Whereas the log cfu count and average DNA concentration of beneficial bacteria (*Lactobacillus*) was significantly increased in PFA and SCFP group, but not in AGP group. The virulence gene expression of the pathogens was down regulated in PFA and SCFP group. The duodenal and jejunal villi height was significantly higher in PFA and SCFP groups, although ratio of villi height to crypt depth in the duodenum, jejunum and ileum did not show significant differences among the groups. On day 28, antibody titers against the IBD and NDV were significantly higher in SCFP groups than other groups. Comparing all the parameters meticulously, it is observed that addition of SCFP at 1.25 kg/MT or PFA at 500 g/MT in diet could produce better nutrient utilization ability, reduction of pathogens and their virulence gene expression, increased *Lactobacillus* count, but incorporation of SCFP increased antibody titre also against two deadly poultry viral infections, NDV and IBDV.

### Institutional review board statement

The study protocol was approved by Intuitional Animal Ethics Committee of West Bengal University of Animal and Fishery Sciences (Ethics Approval Number: 763/GO/Res/ReRc-L/03/CCSEA/67/2023-24), Kolkata, India.

### Informed consent statement

Not applicable.

## Funding

The authors declare that this study received funding from United States Agency for International Development (USAID) under the Transformational Strategies for Farm Output Risk Mitigation (TRANSFORM) Cooperative Agreement (Grant No. TR-SUBAG-FAA-HNR-2023-05) through the Company (Cargill Inc.). The funder was not involved in the study design, collection, analysis, interpretation of data, the writing of this article or the decision to submit it for publication.

## CRediT authorship contribution statement

**Mahamudul Hasan Khan:** Writing – original draft, Validation, Resources, Methodology, Data curation. **Stephen Soren:** Methodology. **Ruma Jas:** Methodology, Formal analysis. **Samiran Mondal:** Methodology, Formal analysis. **Joydeep Mukherjee:** Methodology, Formal analysis. **Manik Chandra Pakhira:** Supervision, Methodology. **Aditya Paul:** Resources, Methodology. **Indranil Samanta:** Writing – review & editing, Validation, Supervision, Data curation, Conceptualization. **Anjan Mondal:** Supervision, Project administration, Funding acquisition, Conceptualization. **Victor Nsereko:** Writing – review & editing, Supervision, Project administration, Funding acquisition, Conceptualization. **Guru Prasad Mandal:** Writing – original draft, Project administration, Funding acquisition, Formal analysis, Conceptualization.

## Disclosures

GPM received the fund from USAID through Cargill Inc. VN and AM are employed by Cargill Inc. but are responsible for USAID funded research and are thus prohibited from participating in any profit-driven Cargill work. The remaining authors declare that the research was conducted in the absence of any commercial or financial relationships that could be construed as a potential conflict of interest. The authors declare that they have no known competing financial interests or personal relationships that could have appeared to influence the work reported in this paper.
